# SNiPlay: a web-based tool for detection, management and analysis of SNPs. Application to grapevine diversity projects

**DOI:** 10.1186/1471-2105-12-134

**Published:** 2011-05-05

**Authors:** Alexis Dereeper, Stéphane Nicolas, Loïc Le Cunff, Roberto Bacilieri, Agnès Doligez, Jean-Pierre Peros, Manuel Ruiz, Patrice This

**Affiliations:** 1 Diversity, Genetics and Genomics of grapevine, UMR DIAPC 1097, INRA, Montpellier, France; 2 Integration of Data, UMR DAP 1096, CIRAD, Montpellier, France

## Abstract

**Background:**

High-throughput re-sequencing, new genotyping technologies and the availability of reference genomes allow the extensive characterization of Single Nucleotide Polymorphisms (SNPs) and insertion/deletion events (indels) in many plant species. The rapidly increasing amount of re-sequencing and genotyping data generated by large-scale genetic diversity projects requires the development of integrated bioinformatics tools able to efficiently manage, analyze, and combine these genetic data with genome structure and external data.

**Results:**

In this context, we developed SNiPlay, a flexible, user-friendly and integrative web-based tool dedicated to polymorphism discovery and analysis. It integrates:

1) a pipeline, freely accessible through the internet, combining existing softwares with new tools to detect SNPs and to compute different types of statistical indices and graphical layouts for SNP data. From standard sequence alignments, genotyping data or Sanger sequencing traces given as input, SNiPlay detects SNPs and indels events and outputs submission files for the design of Illumina's SNP chips. Subsequently, it sends sequences and genotyping data into a series of modules in charge of various processes: physical mapping to a reference genome, annotation (genomic position, intron/exon location, synonymous/non-synonymous substitutions), SNP frequency determination in user-defined groups, haplotype reconstruction and network, linkage disequilibrium evaluation, and diversity analysis (Pi, Watterson's Theta, Tajima's D).

Furthermore, the pipeline allows the use of external data (such as phenotype, geographic origin, taxa, stratification) to define groups and compare statistical indices.

2) a database storing polymorphisms, genotyping data and grapevine sequences released by public and private projects. It allows the user to retrieve SNPs using various filters (such as genomic position, missing data, polymorphism type, allele frequency), to compare SNP patterns between populations, and to export genotyping data or sequences in various formats.

**Conclusions:**

Our experiments on grapevine genetic projects showed that SNiPlay allows geneticists to rapidly obtain advanced results in several key research areas of plant genetic diversity. Both the management and treatment of large amounts of SNP data are rendered considerably easier for end-users through automation and integration. Current developments are taking into account new advances in high-throughput technologies.

SNiPlay is available at: http://sniplay.cirad.fr/.

## Background

The combination of high-throughput re-sequencing and genotyping technologies with the availability of reference genome sequences allows Single Nucleotide Polymorphisms (SNPs) and insertion/deletion (indels) to be extensively characterized in many plant species [1-3], including in grapevine [4-6]. Since the release of the complete sequence of grapevine genome [7,8], the number of large-scale grapevine genetic diversity projects is increasing rapidly, urging the need for automatic polymorphism detection and polymorphism data exploitation. Designing bioinformatics tools able to manage and analyze SNP data in a comprehensive way is therefore essential.

Two large projects have recently been launched: one to produce a SNP diversity map of the entire grapevine genome and the other to estimate linkage disequilibrium between SNPs in four genomic regions. These grapevine projects are based on the present ability to obtain high-throughput sequences and subsequent SNP genotypes using the recently available complete genomic sequence.

In this context, our objective was to conceive an original web-based tool, called SNiPlay, dedicated to polymorphism discovery and analysis in genetic diversity studies. It is an integrated pipeline freely accessible through the internet that combines and transparently chains existing softwares to compute various statistical indices and generate graphical layouts of SNP data. Given standard multiple sequence alignments, Illumina chip genotyping data or Sanger sequencing traces as input, SNiPlay detects SNPs and indels events. In a second round, it sends sequences and genotyping data into an integrative pipeline executing a series of modules in charge of various types of post-processing of the collected polymorphisms and associated genotyping data.

In addition, a database has also been designed and developed to store grapevine polymorphisms, genotyping data and sequences produced by nationally-funded public projects.

To our knowledge, there is to date no freely available web-based tool able to integrate SNP management with as many analytical tools.

Previous software packages have been implemented to automatically discover SNP markers, but most of them are not available through a web server and must be installed on a local computer. Arlequin [9] is a software package for population genetics analysis that integrates several approaches, but it does not include SNP detection. More recently, Anithakumari et al. [10] developed a pipeline for SNP high-throughput detection and mining from EST databases for analysis on the Illumina GoldenGate genotyping platform, and Wegrzyn et al. developed a pipeline for SNP identification from re-sequencing data [11]. HaploSNPer [12], based on QualitySNP [13], is a web-based tool for SNP and allele detection and haplotype reconstruction, but it does not extend the analysis to diversity, linkage disequilibrium or haplotype network study.

SNiPlay aims to fulfil this need as a web application assisting biologists in extracting and analyzing polymorphism data in a simple and robust way, while offering "one-stop-shopping" among leading methods corresponding to well-accepted standards.

The production by the pipeline of well-formatted output data enables their subsequent deeper analysis using external specialized tools.

Since it computes standard FASTA alignments, the pipeline is generic enough to manage sequencing data obtained from traditional Sanger technology as well as those from New Generation Sequencing (NGS) techniques.

## Implementation

SNiPlay combines two major components in a common web interface: a set of pipelined programs and a relational database.

The pipeline consists of Perl modules wrapping various existing programs, except for SNP detection from alignments, which is computed by a home-made module. The SNiPlay database system uses MySQL. The web interface is implemented in Perl CGI scripts running on an Apache web server. The interactivity is allowed by JavaScript and Ajax technologies.

### Pipeline components

**Figure 1:**
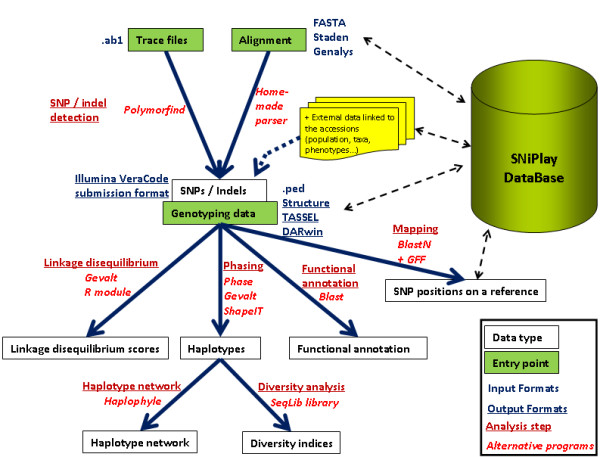
**SNiPlay overview**. This figure illustrates the analysis pipeline implemented in SNiPlay and its relationship with the database. The workflow consists of seven steps: SNP/indel detection, Mapping, Functional annotation, Phasing, Linkage Disequilibrium, Diversity analysis, Haplotype network. The database has been augmented after launching the first two modules of the pipeline.

The automated process consists of seven main modules (Figure 1). The complete process also requires many additional in-house Perl modules (which are not detailed here) to create junctions between the main programs (format compatibility) and to output the results in various formats (e.g. allelic files).

#### SNP/indel detection

This process represents the core of the system, and all subsequent analyses are based on it. Users can start from various entry points using different types of input files and corresponding procedures.

1. When input is provided as standard FASTA alignments, SNiPlay uses a home-made Perl module to detect SNPs and insertion/deletion events and to extract allelic information for each polymorphic position. The detection consists of a simple parsing of the alignment in order to determine positions, i.e. columns, exhibiting variation, either a base variation or a heterozygous IUPAC code for SNP, or the presence of gaps for indel. In a second time, alleles are assigned to genotypes for each variant position. SNiPlay provides both lengths and sequences for indels.

2. When a genotyping file is loaded, a parser extracts allelic data, but the subsequent mapping, annotation and diversity analysis steps are disabled because sequence information is lacking.

3. When the input consists of trace files, the first step makes use of the Polymorfind [14] program, which detects SNPs and indels from PCR product sequencing in heterozygous organisms. Polymorfind itself includes a complete pipeline combining the Phred/Phrap/Consed software suite [15-17] and the Polyscan program [18] for managing polymorphisms. Polymorfind notably has the ability to detect heterozygous indels through a combination of two passes of Polyscan with contrasting stringency.

Whatever the entry point, the next step consists in reporting the statistical information for each polymorphic position, namely, variation, number of alleles, frequency of the most and least frequent alleles, number of readable accessions, and number of homozygous vs. heterozygous accessions for each polymorphism. These SNP statistics are summarized into Excel files available for download. Additionally, this module generates a formatted SNP file for the specific design of Illumina's SNP chips.

Allelic files are generated in various formats so that users can load genotyping data and run locally some widely used external programs dedicated to haplotype reconstruction, association mapping, diversity and phylogenetic analyses and population genetic structure analysis. Indels are binary coded in allelic file so that they can be interpreted as SNP and processed by the phasing programs.

Finally, a FASTA alignment and a consensus sequence showing which nucleotides are most abundant at each position are generated for each set of sequences. This consensus sequence offers a good representation of the initial dataset and will be used subsequently for mapping, functional annotation, and diversity analysis.

#### Mapping to a reference genome

An automatic BLAST-based [19] mapping is then performed using the consensus sequence as the query sequence and a reference genome as the subject. Three complete genomes are currently available (grapevine, sorghum and maize), but other reference genomes may easily be added in the future.

The first best hit is used to localize the amplicon within the genome and a table displays the number of hits to reveal the presence of duplicate genes or pseudo-genes.

The genome-associated GFF3 annotation [20] is then used to obtain the corresponding gene name and structure and then localize polymorphic positions in the corresponding CDS, introns or UTRs. If a SNP is located within a CDS, its sequence is extracted to identify the putative protein and to determine whether the SNP is synonymous. Additionally, the proportion of coding sequence within the amplicon is calculated.

#### Functional annotation

A simple BLAST is performed against the SWISS-PROT and TrEMBL protein databases to further annotate the genomic region and to assign a putative function to the gene. If the mapping reveals that the region corresponds to a predicted gene, the putative protein is then used for BLASTP; otherwise, a BLASTX is launched using the consensus nucleotide sequence as query.

#### Phasing/haplotype reconstruction

A haplotype is the sequence of SNPs in one of the two homologous chromosomes of a specific region, and it cannot be directly established from Sanger sequencing techniques, which are unable to assign each allele to a specific copy within a pair of homologous chromosomes. Haplotype reconstruction consists in inferring the most probable allele combination on each homologous chromosome from genotyping data. Moreover, this method allows missing data to be inferred. The pipeline implements different programs to perform this task, with parameters set at default values: Gevalt [21] using the Gerbil executable [22], PHASE [23] or Shape-IT [24]. As described by Delaneau et al. [24], PHASE is the most accurate program for haplotype reconstruction, but is also the slowest; Shape-IT is the fastest program for small to medium-size SNP samples (< 100 SNPs) and is almost as accurate. Gerbil has been shown to be slightly less accurate but faster when the number of SNPs is low, with the added advantage of defining output haplotype blocks by dynamic programming.

#### Linkage disequilibrium

Two classical measures of linkage disequilibrium (r^2^, D') are defined as the non-random association of alleles between two loci, and their significance can be calculated from the reconstructed haplotypes. HaploView then produces a plot of LD and haplotype blocks along each region/amplicon downloadable as a PNG image.

Alternatively, three new linkage disequilibrium measures correcting for biases due to population structure (r^2^S), relatedness between individuals (r^2^K) or both (r^2^SK) are calculated from genotypic data by a newly adjusted algorithm implemented as an R module [25]. For each pair of markers, estimates of the new r^2^S, r^2^K or r^2^SK measures are provided in addition to the classical r^2 ^measure defined for unphased genotypes using the method of Rogers et al. [26]. The population structure and/or kinship matrices have to be supplied by the user.

#### Diversity analysis

Haplotypes are completed by non-polymorphic bases taken from consensus sequence to obtain complete phased sequences to be analyzed by the SeqLib library [27]. It provides diversity indices including nucleotide diversity (π), number of segregating sites (θ), number of haplotypes (H), haplotype diversity (Hd), and Tajima's D test of neutral evolution [28] for each gene fragment.

If a collection of genes is involved and if mapping has been performed previously, a diversity map is generated to display a color representation of Tajima's D values along chromosomes.

#### Haplotype network and gene phylogeny

The Haplophyle program [29] - based on the "Median Joining Network" algorithm [30] itself linked to the Graphviz graph visualization tool [31] - is used to construct an artificial neural network. Circle size is proportional to haplotype frequency, and the length of connecting lines is proportional to the number of mutational steps between haplotypes. A downloadable PNG image representing a putative phylogenetic network is produced for each gene.

If external information associated with genotypes has been previously specified, the circles can be replaced by colored pies showing the subgroup frequency for each haplotype.

### Pipeline flexibility

#### Input data formats

**Figure 2:**
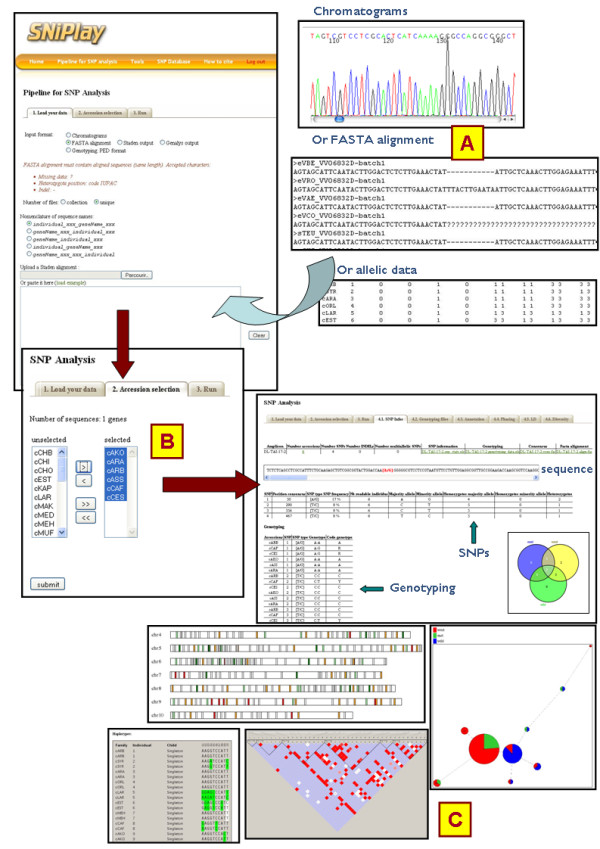
**Overview of the pipeline process and graphical outputs**. (A) Input can be in the form of electrophoregrams, FASTA multiple alignments or genotyping data. (B) Accession selection. (C) Different types of outputs and graphical layouts.

Various input data types and formats are supported depending on the entry point (Figure 2A):

i) Collections of trace files (ABI sequence electrophoregrams). In this case, SNPs and indels are automatically identified using the Polymorfind program.

ii) Alignments in FASTA format (for a given amplicon, each sequence must have the same length). SNiPlay also accepts alignment outputs from the Staden [32] or Genalys [33] programs previously used to align, trim and edit raw Sanger sequencing data.

iii) Genotyping files in the ".ped" format, which is the widely used format for linkage pedigree data required for Gevalt.

Furthermore, alignments or genotyping files can be provided either as a single file or as a zipped collection to analyze several genes in the same run. In order to enable the program to extract gene/amplicon and accession names, users are requested to specify under which form the sequence identifiers are provided.

Electrophoregrams should be provided in a single archive containing all the trace files. If several amplicons are included, each will then be automatically detected using the file nomenclature.

#### Setting parameters and accession selection

Each pipeline step can be set up by choosing among the programs and settings available.

An option is available to first filter out missing data within an alignment. This process removes columns over a certain threshold of missing data (threshold specified by the user), therefore generally eliminating sequence ends with poor trace quality.

In a second tab, users can select subgroups of accessions to be analyzed among those extracted from the input (Figure 2B). SNP discovery will be launched only on the selected subset of genotypes. External data such as population, geographic or phenotypic data, if provided, may also be used to assist in the selection. Moreover, subsequent group comparisons can be obtained by selecting one of the external data fields.

## Results

### Web application

SNiPlay may be accessed at http://sniplay.cirad.fr.

The *SNP pipeline *section constitutes a freely accessible, user-friendly, customizable pipeline with which users can select the analysis steps to be performed, alternative programs being available at some steps. All pipeline results are accessible through a tabulated menu facilitating navigation, and examples are provided to familiarize users with the correct input and expected results (Figure 2C).

The *SNP database *section enables free access to grapevine genetic data managed by our teams and developed from public projects, whereas private projects are password-protected.

All application examples presented below are based on grapevine data because SNiPlay was conceived to satisfy the needs of the grapevine diversity projects. However, SNiPlay is generic enough to be applied to sequence or allelic data of any diploid species.

### SNP database

#### Hosted data types

The database was designed to store SNPs, indels and associated statistical values, genotyping data, sequences, taxa and accessions, SNP genomic locations and annotations.

According to the technology used, the database anticipates two main sources of genotyping data and stores their specificities:

•Sequence data, such as the position in the consensus sequence and flanking regions.

• Illumina VeraCode technology, including OPA name and scoring

It also allows accessions to be linked to predefined characteristics such as population structure, cultivar, phenotype or any criterion that biologists may want to subsequently use as filter for accessions.

#### Database "feeding"

The SNiPlay database has been augmented after launching the first two modules of the pipeline (SNP detection + mapping).

At the time of writing, it includes polymorphisms and genotyping data produced by three public grapevine projects:

• The *Fleshless *project [34] aims at identifying signatures of selection in the region of the "*fleshless berry" *locus.

• A grapevine project used a genome-wide SNP discovery approach based on Sanger re-sequencing of 900 amplicons from 47 accessions: 35 *Vitis vinifera *(including 28 cultivars and 7 wild accessions) and 12 other *Vitis *species. A detailed analysis of these data will be presented in a separate paper.

• A third project concerns polymorphisms in genomic regions involved in berry size determinism.

#### SNP queries

SNiPlay allows one to easily explore data and retrieve SNPs and indels using various filters (such as genomic position, percentage of missing data, polymorphism type, allele frequency, synonymous/non-synonymous, Illumina scores or indel size) to compare SNP patterns between populations and to export genotyping data or sequences in different formats.

**Figure 3:**
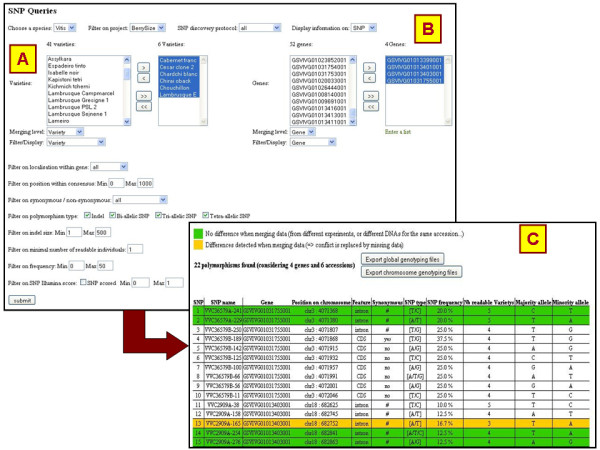
**Web interface for SNP queries**. The system is able to identify SNPs in a user-defined subset of genotypes (A) considering a set of selected genes (B). It can merge allelic data from different origins and different experiments and report polymorphic positions in different colors (C): green if no difference appeared when merging data, yellow if some differences were detected and white if no merging is needed.

After having selected a project, the user has to opt for genotypes on the left side and genomic regions or genes on the right side and adjust the filters; the system then dynamically extracts SNPs for this selection and calculates corresponding frequencies and consensus sequences (Figure 3A). If the subset of genotypes corresponds to the sample initially submitted to the database, pre-calculated and stored SNPs are promptly reported, otherwise they are re-calculated for the selected set of genotypes.

For a SNP with a given position in the genome, the database can collect and combine genotyping data obtained in distinct experiments, from sequencing and/or Illumina VeraCode projects, and helps to manage conflicting data. These positions are highlighted in the results table in green if no difference appears between different experiments or in yellow if differences are detected. In this latter case, the conflict is replaced by missing data when exporting allelic files or calculating SNP frequencies (Figure 3B).

With respect to this concept, the interface can accommodate different "merging levels", from global synthesis (high merging level) to the experimental level (low merging level) using a specific program. The sample can be composed of varieties, accessions or DNA samples, while the sequence can be a gene, an amplicon or a submission batch (as provided by each reader of a given electrophoregram). Depending on the merging level, users can decide to focus on a specific experiment (which will not imply any merging) or force the system to combine allelic data between experiments.

Request results can finally be exported as different files: genotyping file, alignment file or submission file for Illumina VeraCode technology. SNPs with accompanying files can also be exported for submission to GnpSNP, the INRA SNP repository [35].

#### General statistics for a given project

The SNiPlay database currently contains data pertaining to 4 *Vitis *projects for a total of 1,111 amplicons within known genes. The "project overview" option summarizes the data for each project. Today, the database contains 39,163 polymorphisms corresponding to 36,905 distinct positions in the grapevine genome, spanning all the chromosomes and including 2601 indels. Other information that can easily be obtained from the database is the number of mutations in coding vs. non-coding sequences and the number of synonymous vs. non-synonymous substitutions, as well as the average SNP density along the genome.

**Figure 4:**
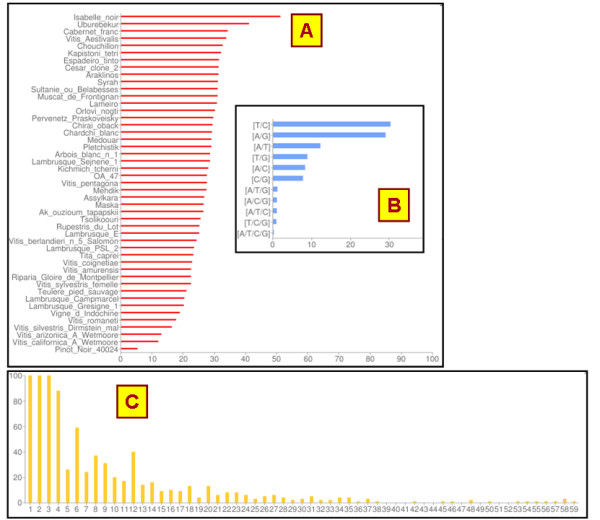
**Examples of global statistics for a given project**. Graphical outputs for a grapevine project (A) Mean heterozygosity of 47 grapevine accessions over 1,111 amplicons (number of heterozygous position over a 10,000 bp region). (B) Frequency of alternative SNP variations. (C) Distribution of indel sizes.

This section also provides graphical outputs comparing the average heterozygosity of accessions, the frequency of alternative nucleotide substitution, and the distribution of indel sizes (Figure 4).

The "group comparison" option allows the user to compare summary data among given sub-populations, for example, among different taxa.

### Benefits and applications

#### Search for selection

**Figure 5:**
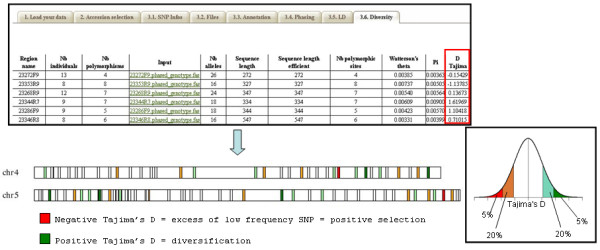
**Diversity map**. Tajima's values are color-coded along the chromosomes. A normal distribution of Tajima's values is assumed to estimate significance: significantly negative values (in red; p < 0.05) suggest an excess of low frequency SNPs, indicating putative regions under purifying selection, while high values (in green) indicate regions where diversification probably occurred.

Adaptation or domestication promotes phenotypic evolution. At genomic level, these changes correspond to genetic variations among targeted loci. Tajima's D index is used to evaluate these modifications and to distinguish between a DNA sequence fitting the neutral theory and one evolving under selection. A negative Tajima's D signifies an excess of low frequency polymorphisms, indicating population size expansion and/or positive selection. A positive Tajima's D signifies a deficit of low frequency polymorphisms, indicating a decrease in population size and/or balancing selection. At the single-locus level, it is impossible to distinguish between demographic effects and selection at this locus. However, analysis of the distribution of Tajima's D for a collection of genes as offered by SNiPlay allows genes targeted by selection to be identified (.i.e., the 5% of genes with the most extreme values) (Figure 5).

#### LD maps

The extent of linkage disequilibrium (LD) around the causal genetic variants responsible for the variation of a trait within an association panel directly determines (i) the ability to identify the genomic region carrying these causal genetic variants given the distribution of genotyped markers spanning the whole genome and (ii) the precision with which causal polymorphisms can be identified, i.e., the minimum physical size of the genomic region carrying the causal variants that are in complete LD with neighbouring ones. Then linkage disequilibrium analysis is generally needed (i) as a prerequisite to whole-genome scanning association studies to determine the number and distribution of markers needed to identify genomic regions involved in phenotypic variation and (ii) a *posteriori *to interpret the results from association studies, notably to determine confidence intervals for the identified genomic regions.

**Figure 6:**
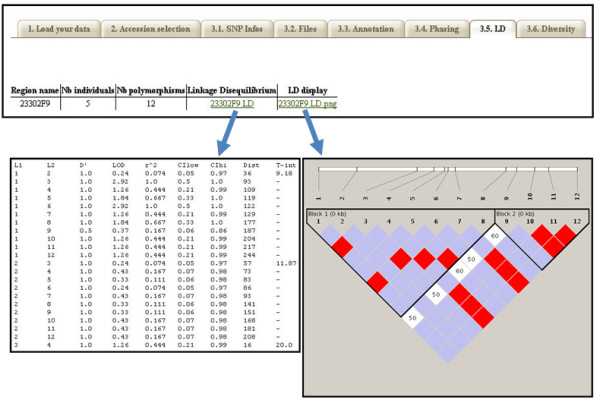
**Haplotype blocks and LD visualization with Haploview**. for each genomic region submitted to SNiPlay, LD scores are calculated for each SNP pairs using the phased genotypes and reported in a LD plot generated by Haploview (via the Gevalt program).

SNiPlay allows two classical pairwise measures of linkage disequilibrium (r^2^, D') and their associated p-values to be calculated from haplotypes inferred from different phasing programs and visualizes them using Haploview (Figure 6). In addition, estimates of three new LD genotypic measures, developed by Mangin et al. [25], that correct for bias due to structure and relatedness were introduced in the pipeline. All these pairwise LD estimates can be exported in tabulated format and used (for instance) to estimate the non-linear relationship between pairwise LD and physical or genetic distances between loci using the models proposed by Sved et al. [36] or Hill et al. [37].

#### Diversity maps

Coupled with medium- or high-throughput technologies such as genotyping arrays, SNP markers are useful tools for studying genetic diversity, genetic structure [38,39], synteny and evolution [40,41]; for establishing genetic maps [42] and association maps [43]; and, when sufficiently dense, for whole genome selection [44]. The SNiPlay pipeline allows one to display maps of SNP frequency or heterozygosity along a genomic region or a whole chromosome and, consequently, to compare values between regions or taxa.

A project aiming at discovering new SNPs in the grapevine genome has utilized SNiPlay to report SNP density, allele frequency, diversity and observed heterozygosity along chromosomal regions. From these results, it was possible i) to define a set of well-distributed SNPs to be used as a reference for other studies (Bacilieri pers. comm.), such as the estimation of kinship for the correction of LD measures, and ii) to allow the specific design of Illumina arrays for other projects focusing on association mapping, for example.

#### Comparison of populations

**Figure 7:**
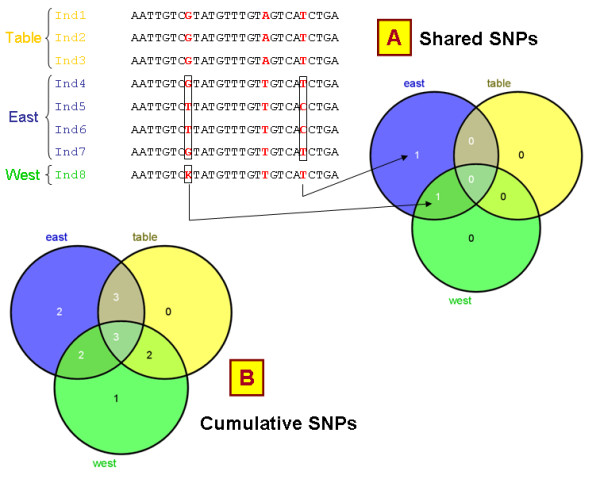
**Venn diagrams**. SNiPlay reports 2 kinds of Venn diagrams indicating (A) the number of polymorphisms (SNPs + indels) shared between groups and (B) the cumulative number of polymorphisms when combining groups.

**Figure 8:**
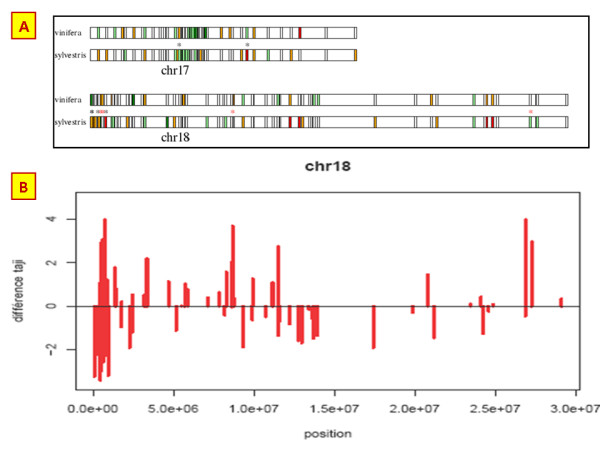
**Example of comparative diversity maps**. (A) A comparison of Tajima's D values over chromosomes 17 and 18 between cultivated and wild grapevine. An asterisk indicates genes for which the difference in Tajima's D value between groups is significant. This map comparison is possible only between the first 2 groups. (B) A genomic region near a "berry size" QTL displays differential D values, which are being further investigated to test for potential association with the wild-cultivated berry size differential.

**Figure 9:**
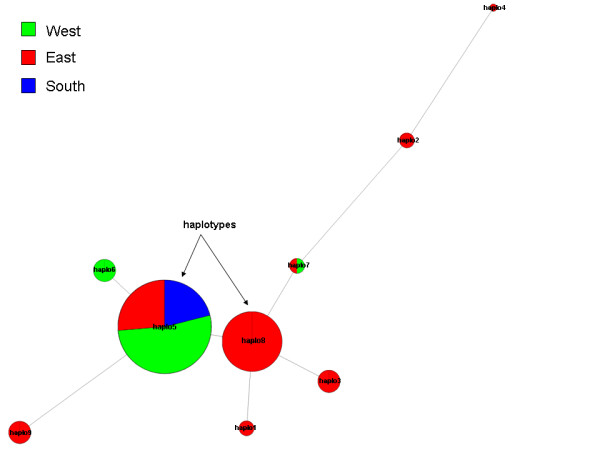
**Example of a haplotype network**. The sizes of the circles are proportional to haplotype frequency, and the lengths of the connecting lines are proportional to the number of mutational steps between haplotypes. External information associated with the genotypes (south, east and west) was specified by the user so that the circles are replaced by colored pies showing the frequency of each subgroup.

In the pipeline section, an optional tabulated file can be provided by the user with external information for each accession such as its wild or cultivated status, its geographical origin, its taxon, population stratification, phenotypic characteristics, or any other information linked to the accessions. This information is taken into account at different levels of the analysis to compare groups or populations:

• Venn diagrams are displayed to report the number of SNPs shared between the different groups (Figure 7A) as well as the number of SNPs newly formed when crossing groups (Figure 7B). Common or group-specific SNPs can then be retrieved in distinct files generated for each diagram section. The Venn diagrams are computed using BioConductor [45] limma package and displayed using Venny [46].

• A comparative diversity map can be generated to show a colour distribution of Tajima's D values along chromosomes for each group (Figure 8). SNiPlay makes it possible to compare different taxa to search for discriminant markers that could facilitate genotype classification into taxa (species, cultivated/wild) and reveal evolutionary patterns.

• The haplotype network integrates coloured pies indicating the distribution of subgroups within each haplotype (Figure 9).

#### Other possible applications invoked using output files

Thanks to the well-formatted outputs offered by SNiPlay, users are able to load "ready-to-use" allelic files and locally invoke well-known external programs for specific purposes: PHASE [23], TASSEL [47], DARwin [48] and STRUCTURE [49], respectively dedicated to haplotype reconstruction, association studies, diversity and phylogenetic analyses and population genetic structure analysis.

### Server limitations

Input limitations depend on the entry point in the pipeline. The server currently accepts archives containing a maximum of 4,000 alignment files for a single run, themselves limited to 200 sequences/accessions. In the case of ABI sequence electrophoregrams, loading is restricted to archives of 2,000 ABI trace files. Allelic files are also limited to a maximum of 500 SNPs and 500 accessions. These limitations could be relaxed in the future, should the application migrate onto more powerful servers.

## Conclusions and future developments

SNiPlay is a flexible and user-friendly web application that rapidly explores SNP data using a wide range of analyses.

Our experiments within the grapevine genetics projects showed that SNiPlay allows geneticists to efficiently manage and analyze the large amounts of SNP data generated by high-throughput technologies and, therefore, to rapidly obtain advanced results in several key areas of plant genetic diversity research.

The major advantage of SNiPlay is that it saves considerable time by automatically detecting SNPs and indels from sequencing data and by injecting allelic data into a reliable multi-analysis system.

It presents detailed and well-organized information so that scientists can avoid routine data processing work and focus on the biological aspects of their own projects. For instance, submitting multiple datasets makes it easy to quickly identify and localize signals of positive selection with the help of diversity maps.

Currently, besides the International HapMap project [50] in Humans, few published databases are dedicated to SNPs and allelic data in other species. FlySNPdb [51] provides SNP data for the major chromosomes of *Drosophila melanogaster*. Recently, AutoSNPdb [52] has been developed to identify and store SNPs from assembled EST sequences in rice, barley and *Brassica *species.

With more than 1,400,000 SNP alleles at 39,163 sites, SNiPlay hosts and provides the grapevine community with a sizeable resource of polymorphisms and other genetic data, and could become the international reference as a grapevine SNP datasource. It can thus provide significant global statistical information on SNPs and indels across the grapevine genome such as density, proportion of synonymous SNPs, and heterozygosity.

SNiPlay is fully functional and currently used by multiple laboratories. Initially conceived to meet grapevine research needs, it was successfully used with grapevine Sanger sequencing data for SNP discovery and genome-wide diversity analysis and with Illumina VeraCode genotyping data for LD fine study in regions of interest. However, SNiPlay provides a generic system for SNP management designed to be applicable to sequence or allelic data of any diploid species. Indeed, the system presently manages allelic data on rice, and it is also being used for *de novo *transcriptome sequencing in various *Coffea *species.

In addition, SNiPlay is independent of the sequencing technology since it accepts standard FASTA alignments. Short reads from NGS deep sequencing experiments (454, Solexa) can make use of SNiPlay, if the assembly have been preliminarily pre-processed and formatted into FASTA alignment files including IUPAC symbols at heterozygous positions. SNiPlay, being a web application, cannot afford such a task online. However, among the further developments planned for coming years, an additional and independent module will be incorporated to pre-process the assembly of short reads from NGS technologies, by detecting SNPs using depth and quality criteria and converting assemblies into FASTA alignments. As a rule, efforts will be constantly undertaken to take into account future technologies.

The second major development planned is the addition of a new component for association studies based on the TASSEL software and/or the GenABEL library [53].

The modular architecture of the SNiPlay pipeline renders this system easily extensible, which will facilitate the addition of new features and programs according to user feedback. For instance, we plan to integrate another phasing program such as FastPhase [54] and to improve the linkage disequilibrium module.

More generally, we intend to integrate in the future new modules able to manage other types of analyses such as kinship, population structure, and phylogeny.

Finally, because SNiPlay is being accepted by the local community working on diversity projects as the tool for SNP management and analysis, it will naturally benefit from continuous maintenance and improvements. It is expected to evolve towards an open-source distribution package including both pipeline and database so that it can be locally and independently installable in multiple labs and adapted for small user communities working on different plant species.

## Availability and requirements

• Project name: SNiPlay

• Project home page: http://sniplay.cirad.fr

• Operating system: web access

• Programming language: PERL

• Any restrictions to use by non-academics: none

## Authors' contributions

ADe wrote the application and coordinated the development. SN and LLC helped to design the software architecture. RB, ADo and JPP helped to define appropriate methods and user needs. MR and PT provided advice and guidance throughout the project.

All the authors read and approved the final manuscript.
